# Supplementary material for: Splice-Junction-Based Mapping of Alternative Isoforms in the Human Proteome
Source: Cell Rep. Author manuscript; Available in PMC 2020 Jan 15. (PMC6961840; doi:10.1016/j.celrep.2019.11.026)
Supplement: 2 [file NIHMS1546469-supplement-2.zip › DF1/PXD009737/TestisTrypsin/TestisTrypsin_21_CEP164_TALSLLGLGEETNEEDEEESDNQESLR.pdf]

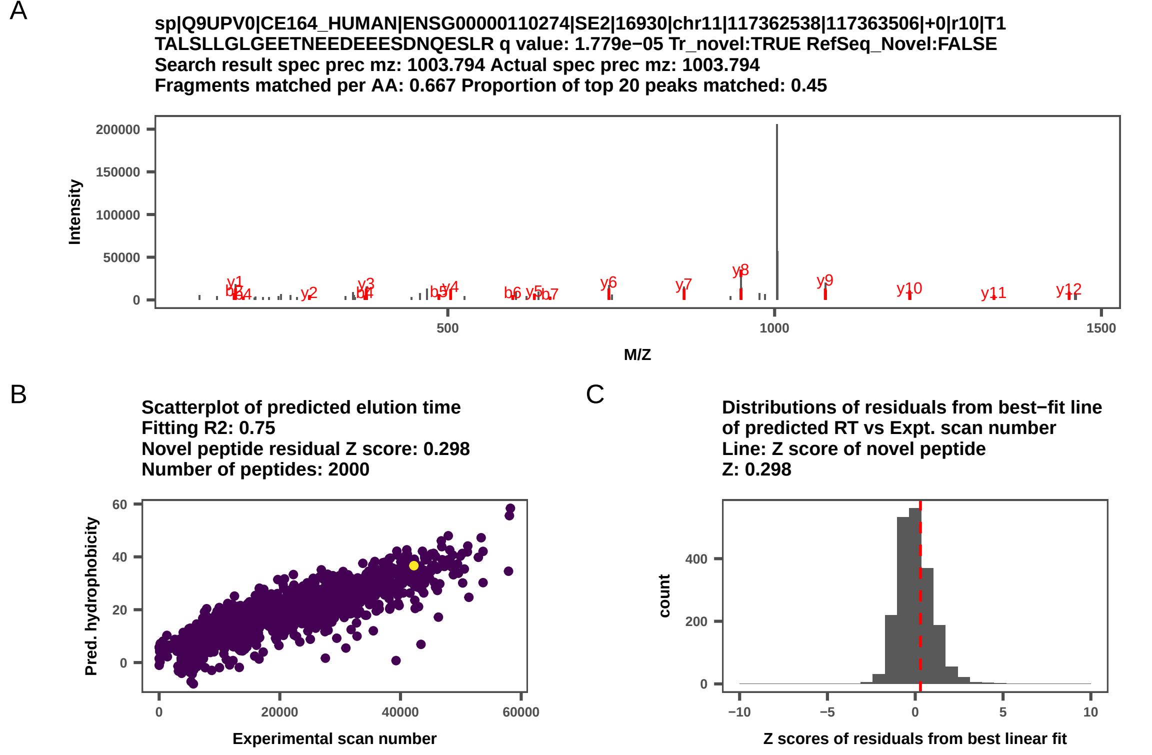
A
sp|Q9UPV0|CE164_HUMAN|ENSG00000110274|SE2|16930|chr11|117362538|117363506|+0|r10|T1
TALSLLGLGEETNEEDEEESDNQESLR q value: 1.779e−05 Tr_novel:TRUE RefSeq_Novel:FALSE
Search result spec prec mz: 1003.794 Actual spec prec mz: 1003.794
Fragments matched per AA: 0.667 Proportion of top 20 peaks matched: 0.45
Intensity
200000
150000
100000
50000
0
y1
y2
y3
y4
b5
b6
y5
b7
y6
y7
y8
y9
y10
y11
y12
500
1000
1500
M/Z
B
Scatterplot of predicted elution time
Fitting R2: 0.75
Novel peptide residual Z score: 0.298
Number of peptides: 2000
Pred. hydrophobicity
60
40
20
0
20000
40000
60000
Experimental scan number
C
Distributions of residuals from best−fit line
of predicted RT vs Expt. scan number
Line: Z score of novel peptide
Z: 0.298
count
400
200
0
−10
−5
0
5
10
Z scores of residuals from best linear fit
